# Elevated *KLF7* levels may serve as a prognostic signature and might contribute to progression of squamous carcinoma

**DOI:** 10.1002/2211-5463.12912

**Published:** 2020-07-13

**Authors:** Jingrun Yang, Kuixia Xie, Zihui Wang, Chengxin Li

**Affiliations:** ^1^ Department of Dermatology PLA General Hospital Beijing China; ^2^ Dermatological Department Tianjin Fifth Centre Hospital Tianjin China; ^3^ Department of Pharmacy Beijing Chao‐Yang Hospital Capital Medical University Beijing China

**Keywords:** carcinogenesis, immunohistochemical staining, KLF7, prognosis, squamous carcinoma, TCGA

## Abstract

Global efforts have been undertaken to define the genome‐wide distribution of epigenetic markers in cancerous tissues, which provide an invaluable opportunity to understand cancer biology and identify predictive signatures. Several studies have focused on the gene expression patterns of squamous carcinoma to identify tumor subtypes and find prognostic and therapeutic targets because squamous carcinoma genomes showed high instability. However, the number of reliable reports referring prognostic significance of genes and their role in squamous carcinoma is still quite limited. Krüppel‐like factor 7 (KLF7) is a transcription factor that is widely expressed in numerous human tissues at low levels. Members of the KLF family have established roles in tumor cell fate, stress response, cell survival and the tumor‐initiating properties of cancer stem‐like cells. Hence to investigate whether *KFL7* expression from cancer tissue holds promise as a prognostic and/or therapeutic target, we analyzed gene expression profiles from squamous carcinoma and surgical margin tissues in The Cancer Genome Atlas. We identified significant up‐regulation of *KLF7* in squamous carcinoma, which was confirmed by immunohistochemical staining. Elevated *KLF7* expression was associated with poor squamous carcinoma prognosis before and after correcting for confounding factors by multivariate Cox regression analysis. Several pathways, such as Neurotrophin and GnRH pathways, were activated in *KLF7*‐up‐regulated squamous carcinoma samples through Gene Set Enrichment Analysis. In conclusion, we consolidate the potential role(s) of *KLF7* in squamous carcinoma carcinogenesis from The Cancer Genome Atlas surgical margin tissue, offering insights into expression signatures that are potentially useful for prognosis modalities.

## Graphical abstract

Here, we evaluated the potential of Krüppel‐like factor 7 (KLF7) as a prognostic signature in squamous carcinoma. Our results suggest that KLF7 is expressed at a high level in squamous carcinoma samples and is associated with poor prognosis. Moreover, our data suggest that several pathways, such as the Neurotrophin and GnRH pathways, are activated in squamous carcinoma samples with high levels of *KLF7*.

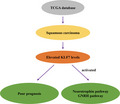

**Abbreviations**
DEG: differential expression gene
EMT: epithelial‐to‐mesenchymal transition
FC: fold change
GnRH: gonadotropin‐releasing hormone
GSEA: Gene Set Enrichment Analysis
IOD: integrated optical density
KEGG: Kyoto Encyclopedia of Genes and Genomes
KLF: Krüppel‐like factor
NC: negative control
OE: overexpression
RT: room temperature
RT‐qPCR: quantitative real‐time polymerase chain reaction
TCGA: The Cancer Genome Atlas

Squamous carcinoma is a tumor that originates in the epithelial cells of the mucosal linings from the upper airway and food passages (including the oral cavity, oropharynx, larynx and hypopharynx). The current established squamous carcinoma risk factors include smoking, excessive alcohol use and high‐risk human papillomavirus infection [1]. Recently, mapping projects have been executed to define the genome‐wide distribution of epigenetic markers in cancerous tissues, which provide an invaluable opportunity to understand cancer biology and identify predictive signatures [2, 3]. Because squamous carcinoma genomes showed high instability [4], more studies have focused on the gene expression patterns of squamous carcinoma to identify tumor subtypes and find prognostic and therapeutic targets [5, 6, 7]. However, the number of reliable reports referring prognostic significance of genes and their role in squamous carcinoma was still quite limited [8].

Krüppel‐like factors (KLFs) are a family of evolutionarily‐conserved transcription factors that are composed of 17 distinct zinc‐finger transcription factors [9]. Members of the KLF family have the capacity to regulate transcription through DNA binding with specificity proteins, which is demonstrated to be involved in tumor cell fate, stress response, cell survival and the tumor‐initiating properties of cancer stem‐like cells [10, 11]. Rajan *et al*. [12] reported that KLF6 seemed to be associated with the biological heterogeneity in prostate cancer. Moreover, studies have shown that KLF4 may act as an oncogene in colon cancer and breast cancer through inhibiting epithelial‐to‐mesenchymal transition (EMT) and cancer stem cell behaviors [13, 14], while the expression of KLF4 was also found to be inversely correlated with increasing tumor stage and grade in breast cancer [15]. Research revealed that high KLF7 level was significantly associated with poor prognosis in lung and gastric cancers [16, 17]. Therefore, the role of KLF7 during the development of squamous carcinoma is not clear.

In this study, we reported the role of KLF7 in squamous carcinoma through comprehensively analyzing gene expression profiles from squamous carcinoma and surgical margin in The Cancer Genome Atlas (TCGA). KLF7 was found to be significantly up‐regulated in squamous carcinoma, and the elevated KLF7 expression was obviously associated with poor squamous carcinoma prognosis. The biological process and signaling pathways regulated by elevated KLF7 were investigated by Gene Set Enrichment Analysis (GSEA). These results will be helpful to clarify the role of KLFs in tumor development and screen therapeutic targets of squamous carcinoma.

## Materials and methods

### Study subjects

Samples used for exploration analysis were obtained from TCGA, including 501 squamous carcinoma tissues and 43 surgical margins with full gene expression profiles. Thirty‐four matched squamous carcinoma and surgical margin tissues are used for immunohistochemistry staining, and the clinical data of the patients are shown in Table S1. All study subjects signed written informed consent. The research was approved by the Medical Ethics Committee of PLA general hospital (clinical registration no. S2018‐223‐02) and in accordance with the Declaration of Helsinki.

### Differential expression analysis

The raw read count‐based expression values were first logarithmically transformed (base 2) for normalization. Differential expression genes (DEGs) in the matched squamous carcinoma samples compared with the 43 surgical margins were identified using the DESeq2 Bioconductor package. Significance of the expression difference was defined by the thresholds of |log2FC| > 1 (fold change [FC]) and Benjamini‐Hochberg‐adjusted *P* (*P*_adj_) < 0.05.

### Immunohistochemical staining

Immunohistochemical staining was performed on 5‐µm‐thick sections obtained from formalin‐fixed paraffin‐embedded tissue of the selected cases. The slides were submerged completely in a preheated antigen retrieval solution and boiled for 15 min. After being cooled to room temperature (RT), the slides were washed with PBS for 5 min and treated with PBS containing 0.5% Triton X‐100 for 20 min at RT. Before staining, the slides were pretreated with 0.3% H_2_O_2_ in methanol for 10 min at RT to block endogenous peroxidase activity and were blocked with the blocking buffer (Beyotime, Beijing, China) for 1 h at RT in a humidified black chamber. Then the slides were washed with PBS three times (5 min per wash) and incubated with the primary antibody of KLF7 (1 : 200, sc‐398576; Santa Cruz Biotechnology, Santa Cruz, CA, USA) at 4 °C overnight in the chamber. After being washed with PBS three times, the slides were incubated with the secondary antibody conjugated (1 : 500, A0286; Beyotime) for 30 min at RT in the chamber. Immunoenzymatic reactions were performed with horseradish peroxidase developed with 3,3′‐diaminobenzidine (Beyotime). All slides were counterstained with hematoxylin (Beyotime). Negative controls (NCs) were performed by omitting the primary antibodies. The images were acquired by an upright microscope (DM3000; Leica, Wetzlar, Germany) equipped with a digital camera (DFC450; Leica).

### GSEA

To identify pathways that are activated or repressed in squamous carcinoma by KLF7, we sorted the squamous carcinoma samples in TCGA in decreasing order of KLF7 expression values and selected out the top 10 (KLF7_high) and bottom 10 (KLF7_low) squamous carcinoma samples for GSEA. Gene sets referring to Kyoto Encyclopedia of Genes and Genomes (KEGG) pathways were obtained from Molecular Signatures Database (MSigDB), and expression differences of all genes between KLF7_high and KLF7_low squamous carcinoma samples were used to quantify gene set activity by using GSEA version 3.0. Enrichment *P‐*value was determined by 1000 permutations, followed by Benjamini‐Hochberg adjustment for the control of false‐positive rate.

### Patient samples and cell lines

Squamous carcinoma (SCC9 and CAL27) cells were purchased from the Cell Bank of the Type Culture Collection (Chinese Academy of Sciences, Shanghai, China). Cells were cultured in 1640 medium (Gibco, Grand Island, NY, USA) containing 10% FBS and 1% 100 U·mL^−1^ penicillin and 100 μg·mL^−1^ streptomycin at 37 °C in a humidified atmosphere.

### Plasmids and transient transfection

KLF7 fragments (NCBI accession no.: NM_003709) were obtained by PCR amplification of cDNA collected from SCC9 cells and were cloned into the expression vector pcDNA3.1 (Addgene, Cambridge, MA, USA). Specific siRNA oligonucleotides targeting KLF7 mRNA were designed by GenePharma Co., Ltd. (Shanghai, China). The oligonucleotide sequence was as follows: siKLF7, 5′‐UCU CGG GAC AAG UUG CUA UTT‐3′ and 5′‐AUA GCA ACU UGU CCC GAG ATT‐3′. Cell transfection was carried out using Lipofectamine™ 3000 (Thermo Fisher Scientific, Inc., Waltham, MA, USA). SCC9 and CAL27 cells were seeded into six‐well plates at ~80% confluence and transduced with Lipofectamine 3000 and plasmid/siRNA to achieve the overexpression (OE) or knockdown of KLF7, respectively. Transfected cells were harvested after 48 and 72 h of transfection for RNA extraction.

### Quantitative real‐time polymerase chain reaction

Total RNA from cells was extracted using TRIzol reagent (Invitrogen, Thermo Fisher Scientific, Inc.) according to the manufacturer’s instructions. RNA was transcribed into cDNA by PrimeScript RT Master Mix (Takara Bio, Inc., Otsu, Japan). Quantitative real‐time polymerase chain reaction (RT‐qPCR) assays were carried out by ABI 7500 real‐time PCR system using TransStart® Top Green qPCR SuperMix (TransGen, Beijing, China). The following primer sequences were used for RT‐qPCR: KLF7, forward, 5′‐ATG GCA CGG TGA CGT TGA AAC T‐3′ and reverse, 5′‐CTC TGG TGG GCC TTT AAG TGG G‐3′; gonadotropin‐releasing hormone (GnRH), forward, 5′‐GGC CTT ATT CTA CTG ACT TGG TG‐3′ and reverse, 5′‐AAT CTT CTT CTG CCC AGT TTC C‐3′; and β‐actin, forward, 5′‐ACC GCG AGA AGA TGA CCC AGA T‐3′ and reverse, 5′‐TCT TTG ATG TCA CGC ACG ATT T‐3′. RT‐qPCR thermocycling conditions were as follows: 94 °C for 30 s, one cycle; at 94 °C for 5 s and 60 °C for 30 s, 40 cycles. Relative mRNA expression levels were measured using the $2^{‐\Delta \Delta C_{t}}$ method [18] and normalized to the internal reference gene β‐actin. All experiments were performed in triplicate.

### Statistical analysis

Comparison of KLF7 expression values between squamous carcinoma and surgical margin was performed by Mann–Whitney *U* test. Kaplan–Meier method was used for the generation of survival curves through the survival package, and significance of difference between survival curves was determined by log rank test. Multivariate Cox regression analysis was used for identifying independent factors for squamous carcinoma prognosis. All of those statistical analyses were conducted using r version 3.4.1 (R Core Team, Vienna, Austria) and bioconductor version 3.1 (http://www.bioconductor.org/). *P*‐value < 0.05 was considered as statistically significant.

## Results

### KLF7 was up‐regulated in squamous carcinoma samples

**Fig. 1:**
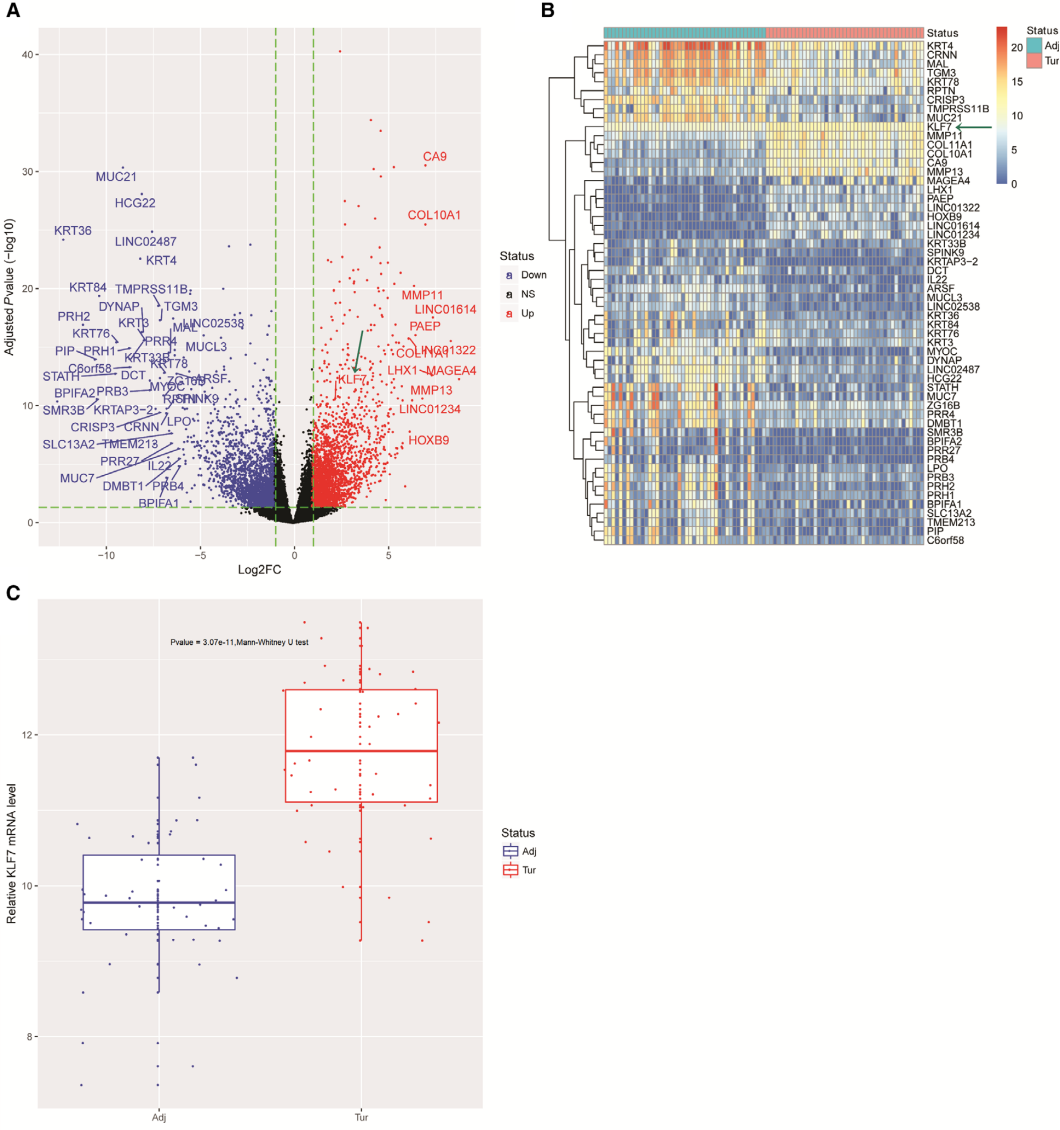
KLF7 was up‐regulated in squamous carcinoma samples. (A) Volcano plot shows the overall differential expression landscape in squamous carcinoma samples (*n* = 43) compared with matched surgical margin (*n* = 43) from TCGA. KLF7 and genes with |log_2_FC| > 6 and *P*_adj_ < 0.05 were specified by their symbols. (B) Heatmap illustrates expression values of KLF7 and genes with |log_2_FC| > 6 and *P*_adj_ < 0.05 in surgical margin and matched squamous carcinoma tissues from TCGA. Blue and red barcodes at the top of the heatmap represent normal and squamous carcinoma samples, respectively. (C) Boxplot shows distribution of KLF7 expression values across surgical margin and matched squamous carcinoma tissues from TCGA. Adj, adjacent; NS, not significant; Tur, tumor.

Based on the thresholds of |log_2_FC| > 1 and *P*_adj_ < 0.05, a total of 6955 DEGs were obtained in matched squamous carcinoma samples compared with the 43 surgical margins as shown in Fig. 1A. KLF7 (indicated with green arrow) and another 55 genes with |log_2_FC| > 6 (i.e., FC > 64) and *P*_adj_ < 0.05 were identified by their symbols in Fig. 1A. A heatmap illustrated their expression values in the matched squamous carcinoma and surgical margin in Fig. 1B. Mann–Whitney *U* test displayed significantly elevated expressions of KLF7 in squamous carcinoma samples compared with surgical margin (Fig. 1C, *P*‐value = 3.07e−11).

### KLF7 protein level was elevated during squamous carcinoma initiation

**Fig. 2:**
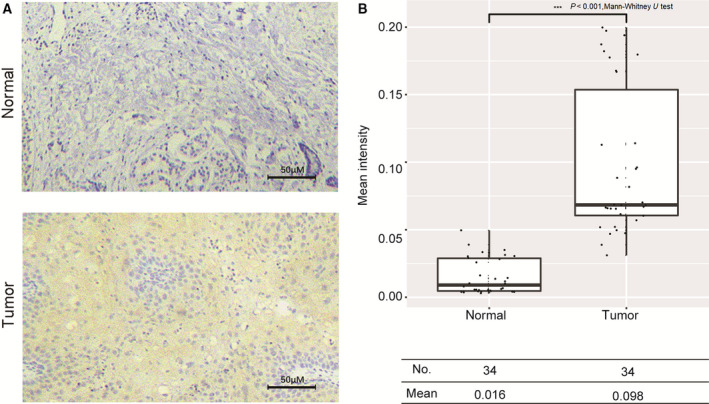
KLF7 protein level was elevated during squamous carcinoma tumorigenesis. (A) Representative plot of KLF7. Immunohistochemical staining in surgical margin and matched squamous carcinoma tissue. Scale bars: 50 µm. (B) Boxplot illustrates the distribution of mean staining intensity across the 34 matched surgical margin and squamous carcinoma tissues calculated via image‐pro plus software. *P < 0.05; **P < 0.01, performed by Mann–Whitney *U* test.

We examined KLF7 protein levels by immunohistochemistry in 34 matched squamous carcinoma and surgical margin tissues. Analysis by image‐pro plus software (Media Cybernetics, Silver Spring, MD, USA) revealed that the KLF7 protein level was significantly elevated in squamous carcinoma samples as shown in Fig. 2A,B and Table S2, which was consistent with KLF7 mRNA expression analysis in matched squamous carcinoma and surgical margin tissues in TCGA.

### KLF7 was an independent squamous carcinoma prognosis signature

**Fig. 3:**
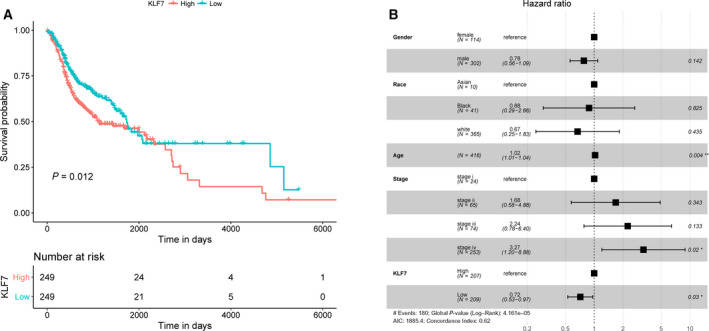
KLF7 was an independent prognosis signature for squamous carcinoma. (A) Overall survival curves of squamous carcinoma samples stratified by median KLF7 expression value generated by the Kaplan–Meier method. *P*‐value was determined by log rank test. (B) Forest plot of multivariate Cox regression analysis indicated KLF7 as an independent and robust biomarker for squamous carcinoma prognosis. The third column represents hazard ratio, and numbers in brackets are 95% confidence interval. The fourth column represents *P‐*values, with *P < 0.05 and **P < 0.01, respectively.

To explore the association between KLF7 expression and squamous carcinoma prognosis, we divided the 498 patients with squamous carcinoma with complete survival information into two groups according to the median KLF7 expression value (11.62) and plotted the overall survival curve for every subgroup using the Kaplan–Meier method as shown in Fig. 3A. Log rank test showed a significant difference between the two survival curves (*P*‐value = 0.012); that is, high KLF7 expression was associated with poor squamous carcinoma prognosis. Multivariate Cox regression analysis determined KLF7 as an independent and robust signature for squamous carcinoma prognosis after correcting for the influences of age, gender, race and stage (Fig. 3B).

### Pathways might be regulated by KLF7 in squamous carcinoma

**Fig. 4:**
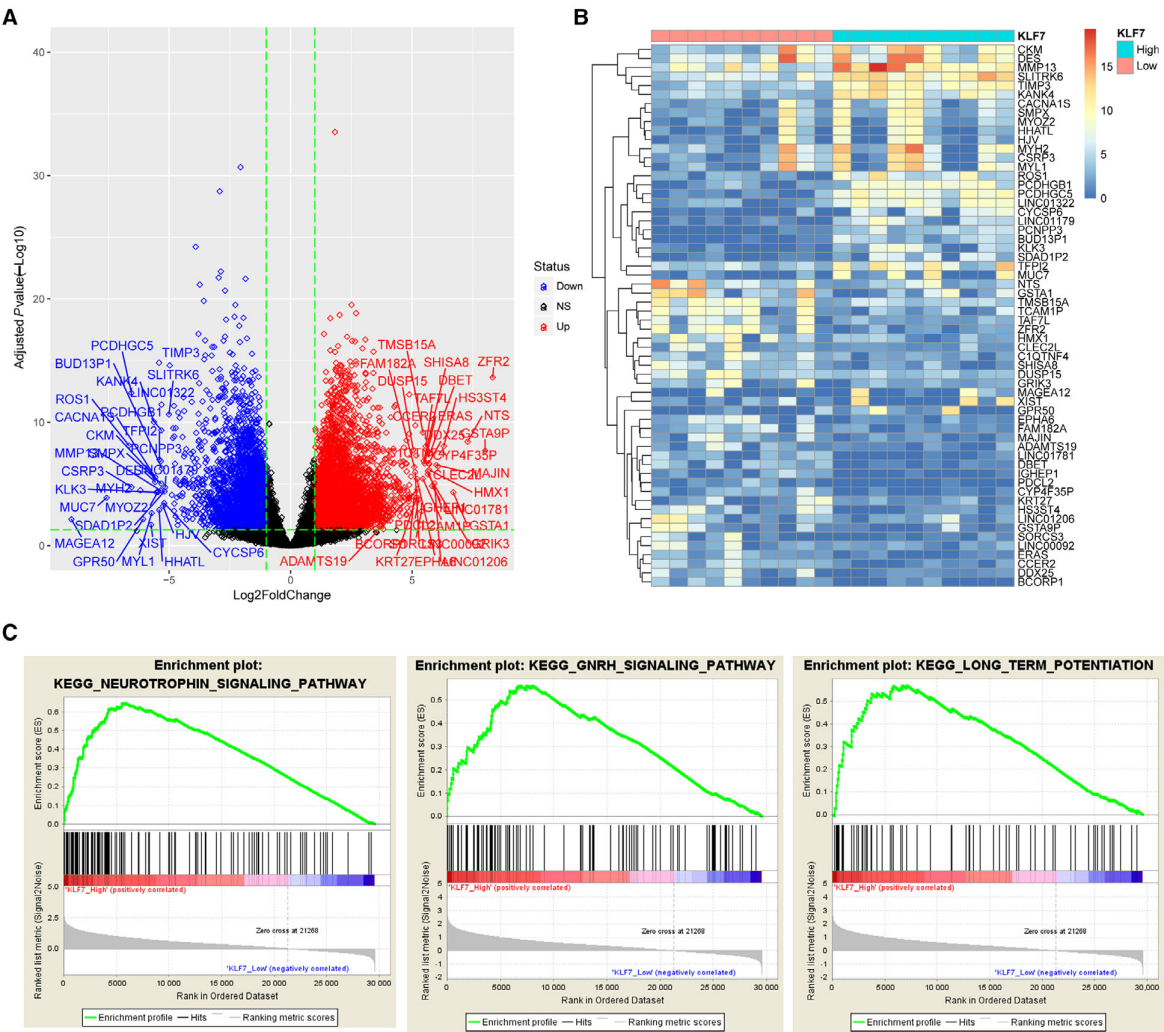
GSEA identified potential pathways that are activated or repressed by KLF7 in squamous carcinoma. (A) Volcano plot shows differential expression landscape in KLF7_high samples (*n* = 251) compared with KLF7_low squamous carcinoma samples (*n* = 250) from TCGA. Genes with |log_2_FC| > 5 and *P*_adj_ < 0.05 were specified by their symbols. (B) Heatmap illustrates expression values of genes with |log_2_FC| > 5 and *P*_adj_ < 0.05 in KLF7_high and KLF7_low squamous carcinoma samples. (C) Details of the top three most significantly enriched KEGG pathways in KLF7_high squamous carcinoma samples. NS, not significant.

We identified 9579 DEGs between KLF7_high and KLF7_low squamous carcinoma samples, out of which 69 genes with |log_2_FC| > 5 (i.e., FC > 32) were identified by their symbols in Fig. 4A. A heatmap illustrated their expression values in KLF7_high and KLF7_low squamous carcinoma samples in Fig. 4B. Protein‐protein interaction analysis of those 69 genes identified a total of 16 interacted gene pairs by STRING online tool (https://string‐db.org/). The protein‐protein interaction network was shown in Fig. S1, which could partially reflect the pathways through the mechanism that KLF7 used to regulate squamous carcinoma carcinogenesis and prognosis.

A total of 19 KEGG pathways were significantly activated or repressed in KLF7_high squamous carcinoma samples according to the thresholds of *P*_adj_ < 0.05 and normalized enrichment score > 1.9 that are provided in Table 1. Details for the top three most significant pathways, including Neurotrophin signaling pathway, GnRH signaling pathway and long‐term potentiation, were shown in Fig. 4C.

**Table 1:** Significantly enriched pathways in KLF7_high head and neck squamous cell carcinoma samples. ERBB, V‐erb‐b2 erythroblastic leukemia viral oncogene homolog; FDR, false discovery rate; Hits, gene counts in pathway; MAPK, mitogen‐activated protein kinase; NES, normalized enrichment score.

Pathway	Hits	NES	FDR
Neurotrophin signaling pathway	124	2.183	0.00107
GnRH signaling pathway	97	2.166	0.00147
Long‐term potentiation	67	2.092	0.00631
ERBB signaling pathway	86	2.052	0.0112
Prostate cancer	88	1.934	0.0121
Adipocytokine signaling pathway	62	1.941	0.0128
SNARE interactions in vesicular transport	38	1.934	0.0129
Regulation of actin cytoskeleton	201	1.944	0.0135
GAP junction	87	2.007	0.0140
Long‐term depression	65	1.977	0.0142
Vasopressin‐regulated water reabsorption	41	1.904	0.0142
Insulin signaling pathway	131	1.961	0.0142
Renal cell carcinoma	66	1.945	0.0146
Focal adhesion	196	1.905	0.0149
Amino sugar and nucleotide sugar metabolism	43	1.954	0.0153
Oocyte meiosis	106	2.021	0.0153
Ubiquitin‐mediated proteolysis	130	1.907	0.0155
Chronic myeloid leukemia	73	1.948	0.0156
MAPK signaling pathway	252	1.980	0.0157

### KLF7 regulated the expression of markers of the GnRH signaling pathway

**Fig. 5:**
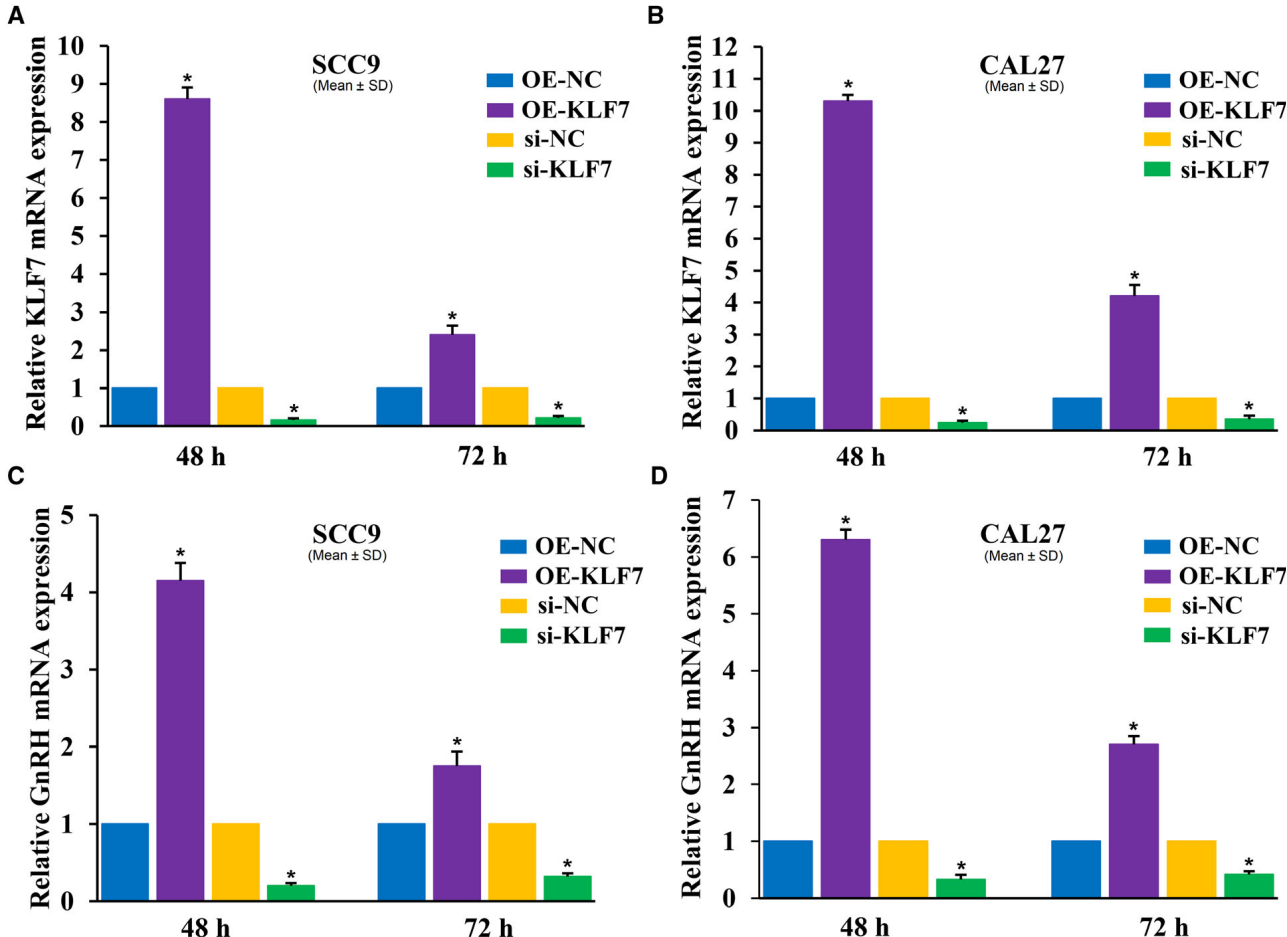
Effect of KLF7 on GnRH mRNA expression. (A) KLF7 mRNA expression in response to OE or knockdown of KLF7 in SCC9 cells after 48‐ and 72‐h transfection. (B) KLF7 mRNA expression in response to OE or knockdown of KLF7 in CAL27 cells after 48‐ and 72‐h transfection. (C) GnRH mRNA expression in response to OE or knockdown of KLF7 in SCC9 cells after 48‐ and 72‐h transfection. (D) GnRH mRNA expression in response to OE or knockdown of KLF7 in CAL27 cells after 48‐ and 72‐h transfection. Data were presented as the means ± SD from three independent experiments. *P < 0.05 compared with the control. si, small interfering RNA.

In order to further confirm that high‐level expression of KLF7 could activate the GnRH signaling pathway, the mRNA expression level of the GnRH signaling pathway marker GnRH was examined in SCC9 and CAL27 cells using RT‐qPCR. The result of transfection efficiency showed that the mRNA level of KLF7 was significantly increased in the OE‐KLF7 group compared with that in the OE‐NC group, but was markedly decreased in the si‐KLF7 group compared with that in the si‐NC group in SCC9 and CAL27 cells after 48 and 72 h of transfection (Fig. 5A,B). The mRNA level of GnRH was obviously enhanced in the OE‐KLF7 group compared with that in the OE‐NC group, but remarkably reduced in the si‐KLF7 group compared with that in the si‐NC group in SCC9 and CAL27 cells after 48 and 72 h of transfection (Fig. 5C,D). Together, these results demonstrated that the GnRH signaling pathway was activated by KLF7 of high level, which was consistent with the earlier result.

## Discussion

Although developed in the same cell type in one tissue, squamous carcinoma is actually a heterogeneous tumor, which is believed to be related to the different etiological and complex changes in gene expression and signaling pathways [4]. In addition to genetic/epigenetic clonal evolution, the cancer stem cell model also provides a mechanism for generating phenotypic and functional heterogeneity in multiple cancers [19]. The transcription factors (*TP63*, *SOX2*) implicated in homeostasis of epithelial differentiation were found to be abnormally expressed in squamous carcinoma [20], and cell cycle, death, nuclear factor‐κB and other oncogenic pathways related to the development of squamous carcinoma. These genetic instabilities were associated with tumor aggressiveness and drug resistance, and finally lead to a poor prognosis [21].

In this study, the expression of KLF7, one of the regulators of cell proliferation and differentiation in several different organ systems, was found to be significantly up‐regulated in squamous carcinoma tissues compared with surgical margin. For epithelial malignancies, including squamous carcinoma, the EMT was defined as the crucial event in the metastatic process, and the markers of EMT were associated with the poor prognosis of squamous carcinoma [22]. In the studies on human squamous carcinoma, KLF7 OE was proved to promote migration and induce EMT and lymph node metastasis through the expression of Snail [23].

The KLFs could respond to the regulation of *Oct4* and *Stat3*, enhancing the pluripotency, identity and self‐renewal of embryonic stem cells [24]. Among them, KLF7 was first reported as an important regulator for neuronal morphogenesis in selected regions of the nervous system [25]. Recently, *KLF7* gene expression was demonstrated to be required for the differentiation of neuroectodermal and mesodermal cells [26]. However, its regulatory role in tumor stem cells has not been reported. In this study, the signaling pathways regulated by KLF7 in squamous carcinoma were identified by GSEA, and the three most significant pathways were Neurotrophin signaling pathway, GnRH signaling pathway and long‐term potentiation. The result of RT‐qPCR also demonstrated that high KLF7 mRNA expression could enhance the GnRH mRNA expression in SCC9 and CAL27 squamous carcinoma. Studies showed that the GnRH pathway was involved in the self‐renewal of lung cancer stem‐like cells through up‐regulating the JNK signaling pathway [27]. Therefore, we speculated that the KLF7/GnRH/JNK pathway might participate in the regulation of squamous carcinoma stem cells.

In addition, signaling pathways associated with neural development and functions (Neurotrophin signaling pathway and long‐term potentiation) were significantly enriched. In squamous carcinoma, perineural tumor growth was a route for cancer extension and was significantly associated with poor prognosis [28]. The cancer cells in a nerve environment not only showed increased proliferation and decreased apoptosis, but also secreted molecules to promote the neurite outgrowth from the nerve toward the tumor [29]. Therefore, it could be speculated that the elevated expression of KLF7 might be able to promote the proliferative and survival behavior of squamous carcinoma cells in the nerve environment and mediate the cellular interactions with nerve.

There are also some limitations in this study. First, the number of samples was relatively small. Second, the pathways involved in KLF7 regulation of squamous carcinoma development remained unclear. In future research, we will integrate the data on multiple platforms, expand the sample size and investigate the molecular regulatory network of KLF7 through cell biology experiments. Finally, KLF7 was found to be broadly expressed at low levels in adult tissues [30], and its expression could be regulated by miR‐185 [31]; this study was expected to contribute to the crafting of epigenetic therapeutic strategies for squamous carcinoma.

## Conflict of interest

The authors declare no conflict of interest.

## Author contributions

JY made substantial contributions to conception and design, acquisition of data, analysis and interpretation of data. KX performed the experiments. ZW has been involved in drafting the manuscript or revising it critically for important intellectual content. JY and KX gave final approval of the version to be published. CL agreed to be accountable for all aspects of the work in ensuring that questions related to the accuracy or integrity of any part of the work are appropriately investigated and resolved.

## Supporting information


**Fig**.** S1**. Protein‐protein interaction network of DEGs between KLF7_high and KLF7_low groups.

**Table S1**. Clinical data for each patient with head and neck squamous cell carcinoma.

**Table S2**. Mean IOD values of normal and tumor tissues.

## Data Availability

The data that support the findings of this study are available in TCGA‐HNSC at (https://portal.gdc.cancer.gov/projects/TCGA‐HNSC). Data are available from the corresponding author on request.
