# Supplementary figures and images for: Elevated KLF7 levels may serve as a prognostic signature and might contribute to progression of squamous carcinoma
Source: FEBS Open Bio. 2020 Jul 13;10(8):1577–86. doi: 10.1002/2211-5463.12912 (PMC7396437; doi:10.1002/2211-5463.12912)

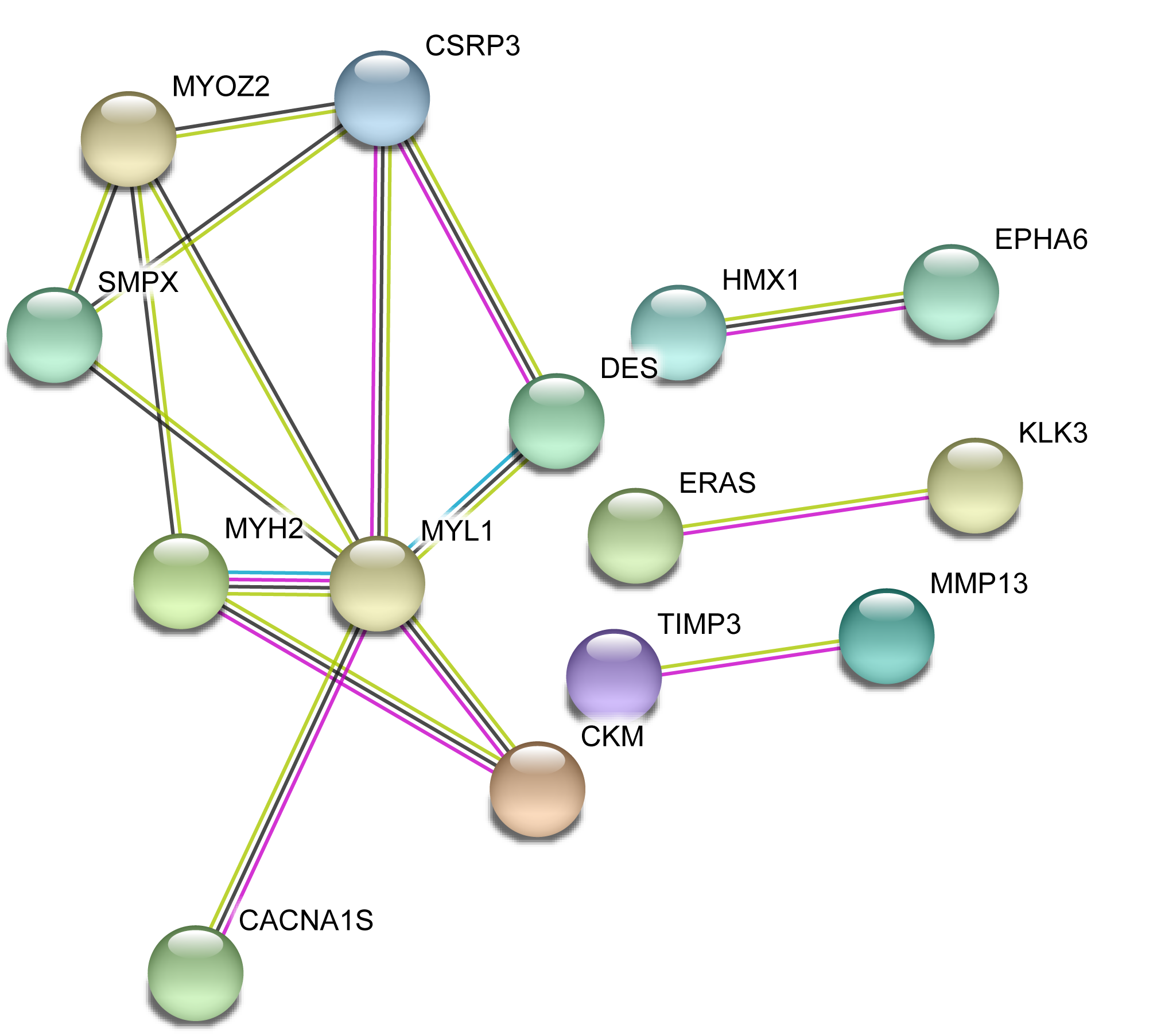

Supplement: Supplementary file 1 — Fig. S1. Protein‐protein interaction network of DEGs between KLF7_high and KLF7_low groups. [file FEB4-10-1577-s001.tif]
